# Gut microbiota in systemic lupus erythematosus patients and lupus mouse model: a cross species comparative analysis for biomarker discovery

**DOI:** 10.3389/fimmu.2022.943241

**Published:** 2022-08-02

**Authors:** Eya Toumi, Benoit Goutorbe, Anne Plauzolles, Marion Bonnet, Soraya Mezouar, Muriel Militello, Jean-Louis Mege, Laurent Chiche, Philippe Halfon

**Affiliations:** ^1^ Aix-Marseille Univ, Microbes, Evolution, Phylogénie et infection (MEPHI), Institut de recherche pour le développement (IRD), Assistance Publique-Hopitaux de Marseille (APHM), Marseille, France; ^2^ Institut Hospitalo-universaire (IHU)-Méditerranée Infection, Marseille, France; ^3^ Laboratoire Alphabio, Clinical Research and R&D Department, Marseille, France; ^4^ Centre de Recherche en Cancérologie de Marseille (CRCM), Aix‐Marseille Univ U105, Inserm U1068, CNRS UMR7258, Institut Paoli‐Calmettes, Marseille, France; ^5^ Université Paris-Saclay, Institut national de recherche pour l’agriculture, l’alimentation et l’environnement (INRAE), Mathématiques et Informatique Appliquées du Génome à l’Environnement (MaIAGE), Jouy-en-Josas, France; ^6^ Hopital de la Conception, Immunology Department, Marseille, France; ^7^ Infectious and Internal Medicine Department, Hôpital Européen Marseille, Marseille, France

**Keywords:** systemic lupus erythematosus, gut microbiota, dysbiosis, disease activity, outcome assessment, health care, biomarkers

## Abstract

An increasing number of studies have provided strong evidence that gut microbiota interact with the immune system and stimulate various mechanisms involved in the pathogenesis of auto-immune diseases such as Systemic Lupus Erythematosus (SLE). Indeed, gut microbiota could be a source of diagnostic and prognostic biomarkers but also hold the promise to discover novel therapeutic strategies. Thus far, specific SLE microbial signatures have not yet been clearly identified with alteration patterns that may vary between human and animal studies. In this study, a comparative analysis of a clinically well-characterized cohort of adult patients with SLE showed reduced biodiversity, a lower *Firmicutes/Bacteroidetes* (*F/B*) ratio, and six differentially abundant taxa compared with healthy controls. An unsupervised clustering of patients with SLE patients identified a subgroup of patients with a stronger alteration of their gut microbiota. Interestingly, this clustering was strongly correlated with the disease activity assessed with the Systemic Lupus Erythematosus Disease Activity Index (SLEDAI) score (*p = 0.03*, odd ratio = 15) and the identification of specific alterations involving the *F/B* ratio and some different taxa. Then, the gut microbiota of pristane-induced lupus and control mice were analyzed for comparison with our human data. Among the six differentially abundant taxa of the human disease signature, five were common with our murine model. Finally, an exhaustive cross-species comparison between our data and previous human and murine SLE studies revealed a core-set of gut microbiome species that might constitute biomarker panels relevant for future validation studies.

## Introduction

Systemic lupus erythematosus (SLE) is a complex autoimmune disease characterized by a breakdown in tolerance to nuclear antigens. This leads to immune-complex deposits that cause severe inflammation in various organs such as the skin, joint, and kidney. Its broad-spectrum manifestations and its unpredictable course between active and remissive stages complicate the disease monitoring and represent a challenge to clinicians (1). SLE primarily affects women of child-bearing age, and its etiology remains unclear but there is strong evidence that genetic, hormonal, and environmental factors are involved (2). Current SLE treatments are mainly immunosuppressive drugs with unsatisfactory clinical response and functional remission rates and can lead to serious side effects (3, 4). Additionally, the long-term use of these treatments has been associated with higher incidences of more severe infections (5). There is now a crucial need to better understand the pathogenesis of SLE and propose a new therapeutic strategy without adverse effects to improve both the quality of life and survival of patients with SLE.

Recently, with the revolutionary advances in next generation sequencing (NGS) technique, emerging investigations in human and murine models have shown that disturbed microbial compositions and functions called “dysbiosis” are involved in the pathophysiology of autoimmune diseases such as inflammatory bowel disease, type 1 diabetes, rheumatoid arthritis, and multiple sclerosis (6). Growing evidence suggests that gut microbiota also play a role in SLE pathogenesis (7–9). A gut permeability called “leaky gut” was observed in lupus studies leading to altered gut barrier function (10). A decrease in beneficial bacteria such as *Bifidobacterium* (11, 12) and an increase in harmful bacteria such as *Enterococcus gallinarum* (13) and *Ruminococcus gnavus* (14), which are closely related to disease progression, were observed in both human and murine lupus. Hence, gut microbiota analysis may offer new possibilities for early diagnosis, prevention, and therapeutic approaches based on gut microbiome modulation in SLE.

However, to date, the association between gut dysbiosis and SLE activity remains unclear. Existing studies are limited to only observational case-control reports in which gut microbiome dynamics are compared to matched controls with a single time-point analysis and therefore a considerable risk of finding false positive associations. Additionally, there is discordance between human cohorts due to differences in the ethnicity and lifestyle of the populations studied. The few existing interventional studies involve only murine models that may differ in anatomy and physiology from human patients with SLE. Currently, there are no comparative studies between the two. Thus, longitudinal studies are needed to establish a common signature of gut microbiota in human and murine SLE that can serve as diagnostic and prognostic biomarkers for the disease.

Through this study, we first longitudinally investigated the dynamics of the gut microbiota of both active and inactive patients with SLE compared with a healthy population. Then, we explored the association between the gut dysbiosis and the disease activity to propose the first French gut microbiome signature of SLE. We further analyzed the murine gut microbiota in a pristane-induced lupus mouse model to identify a common and robust microbial signature of the disease between humans and mice. Finally, based on our results and those of existing studies, we propose a panel of bacterial populations commonly found to define a universal gut microbiota signature of SLE.

## Materials and methods

### Human study design

Stool samples from patients aged ≥18 years with a diagnosis of SLE according to the American College of Rheumatology (ACR) criteria, regardless of disease activity and ongoing treatments, were collected in the European Hospital of Marseille. Disease activity was scored based on the Systemic Lupus Erythematosus Disease Activity Index (SLEDAI) (15). Patients with SLE with severe anemia (Hb <7 g/dl) and pregnancy were excluded. From six months to one year after their first stool sample collection, some of the included patients with SLE have provided a second stool sample for the longitudinal microbiota analysis. Patients with SLE were compared with healthy controls (HCs) recruited by considering the sex-ratio of patients with SLE as well as their age range. These individuals have no known chronic pathology or any specific treatment that could disrupt their gut microbiota during the last two months preceding the stool sampling.

### Animal experimental design and lupus induction

Nine-week-old female BALB/cByJ (Charles River Laboratories, L’Arbresle, Lyon, France) were housed in a controlled temperature and pressure environment. Mice were adapted to new environmental conditions for one week before the beginning of the experimental procedure. The animals were kept in cages with water and food *ad libitum*, enriched with cardboard houses with cotton squares as nests.

Animals were randomly divided into two groups, including a pristane-induced-lupus (PIL) group (n = 5) that received a single intra-peritoneal injection of 500 µl of sterile pristane oil (2, 6, 10, 14-tetramethylpentadecane, Sigma Aldrich, MO, USA) according to Satoh etal. (16) and a control (CO) group (n = 5) that received a single intra-peritoneal injection of 500 µl of sterile phosphate buffered saline (PBS, Sigma Aldrich). Blood, stool, and urine samples were collected before PBS/pristane induction (Day 0) and at six months post-induction (M6). The animals were observed weekly for clinical monitoring. At the end of the experiment (M6), all animals were euthanized by lethal overdose of Dolethal^®^ after an anesthetic protocol (including 90 mg/kg of ketamine^®^ and 10 mg/kg of xylazine^®^).

### Evaluation of SLE-like disease in PIL mice

Immunological and inflammatory analyses were performed in serum samples collected on day 0 and at M6 post-induction to validate the SLE onset. Immunological analysis included antinuclear antibody (ANA) detection determined using the indirect immunofluorescence method using commercial slides containing HEp-2 cells (Kallestad HEp-2 Cell Line Substrate, 12-well slides, Bio-Rad Laboratories, Hercules, CA) and antibodies against double-stranded-DNA (ds-DNA) quantification using the ELISA method using the mouse anti-dsDNA IgG-specific ELISA kit (Mybiosource, San Diego, CA, USA) according to the instructions of the manufacturer. For inflammatory analysis, levels of interferon (IFN)-α (PBL assay science, Piscataway, NJ, USA), tumor necrosis factor (TNF)-α (Aviva System Biology, USA) and C-reactive protein (CRP) (Aviva System Biology, USA) were measured using commercially available ELISA kits according to the instructions of the manufacturer.

A clinical assessment of arthritis was performed weekly starting two weeks after pristane induction, looking for redness and swelling in the paws. Histopathology and immunofluorescence analysis were performed in the kidneys and lungs to investigate tissue damage and immune-complex deposits.

### Stool sample collection and 16S rRNA sequencing

Human and murine stool samples were collected, stored immediately in stabilizing solution (DNA/RNA shield, Zymo Research, Freiburg, Germany) and frozen at −20°C until analysis. Bacterial DNA was isolated using the ZymoBIOMICS DNA prepKit (Zymo Research) following the instructions of the manufacturer. To determine the gut microbiome composition of each sample, a metagenomic sequencing library targeting the V3-V4 regions of the 16S rRNA gene was created following Illumina’s recommendations as previously described (17).

### Bioinformatic processing

Sequencing reads were processed with an in-house pipeline, as previously described (17). Briefly, preprocessing and denoising were performed using Qiime2 (18) (version 2021.11) and DADA2 (19). Resulting amplicon sequence variants (ASVs) were taxonomically assigned with Kraken (20) (version 1.1) based on the NCBI RefSeq Targeted Loci database. Phylogenetic tree of ASVs were generated independently for human cohort and mice experiment was built using mafft (21) (version 7.407) and fast tree (22) (version 2.1.10) with default parameters.

### Statistical analysis

Statistical analysis was performed with R (version 4.1) using the phyloseq package (version 1.36.0) (23). We ensured a minimal depth of 50 000 reads per sample, that discarded 3 of 79 available HC samples (consequently, only 76 HC samples were used for downstream analyses). We performed a rarefaction at lowest sample depth for human and murine data sets independently, resulting in 56 219 reads/sample and 62 463 reads/sample respectively. To compare microbiotas diversity and composition, we assessed alpha-diversity by Shannon index, beta-diversity by Bray-Curtis dissimilarity index which was visualized through principal component analysis (PCoA). Permanova test was performed to track the effect of clinical conditions on distances between samples. Differentially abundant taxa were identified using DESeq2 method (24) (version 1.32.0). Unsupervised classification of samples was performed based on Bray-Curtis dissimilarity indices, using hierarchical clustering with Wards linkage criteria and the two main clustered were retrieved. We then compared clustering results to disease activity and other clinical variables available (see **Supplementary Data** for details).

## Results

### Clinical and biological characteristics of SLE patients

A total of 16 SLE patients and 76 sex-age matched HCs were included. The mean age was 42 ranged from 19 to 70 years old and a female-to-male ratio of 7:1. At inclusion, SLEDAI score ranged from 0 to 12, with 9/16 patients having inactive SLE (SLEDAI=0). For the therapeutic regimen, 12/16, 4/16 and 1/16 patients received hydroxychloroquine, prednisone or immunosuppressants, respectively. Basic clinical and biological characteristics of SLE patients were shown in **Table 1**.

**Table 1:** Clinical and biological characteristics of SLE patients.

ID patient	Age (years)	Sex	BMI	PGA	SLEDAI	Low complement levels	Positive anti-dsDNA titres	AHT	Type 2 diabetes	APS	Ongoing SLE treatments
001	19	M	18.7	0.12	0	no	no	no	no	no	no
002	22	F	22.8	2.1	12	yes	yes	no	no	no	HCQ CT
003	25	F	23.8	0.63	0	no	no	no	no	yes	HCQ
004	49	F	35.1	0	0	no	no	no	no	no	no
005	55	F	17.9	0.3	0	no	no	no	no	no	no
006	33	F	23.1	0	2	no	no	no	no	yes	HCQ AZA
007	70	F	23.3	0.72	2	no	yes	no	no	no	HCQ CT
008	46	F	20.7	0.75	2	no	yes	no	no	no	HCQ
009	69	F	23.1	0.27	0	no	no	no	no	no	CT
011	43	F	24.2	0.09	0	no	no	no	no	no	HCQ CT
012	33	F	21.9	0.21	4	yes	yes	no	no	no	HCQ
013	28	M	18.8	0.24	0	no	no	no	no	no	HCQ
014	55	F	19.3	1.02	0	no	no	no	no	yes	HCQ
017	35	F	19.6	0.66	0	no	no	no	no	no	HCQ
018	49	F	32	0.84	4	no	no	no	yes	no	HCQ
019	38	F	21.7	0.21	2	no	no	no	no	no	HCQ

M, male sex; F, female sex; BMI, Body mass index; PGA, Physician Global Assessment; SLEDAI, SLE Disease Activity Index; AHT, Arterial hypertension; APS, antiphospholipid syndrome; HCQ, hydroxychloroquine; CT, corticosteroids; AZA, azathioprine (immunosuppressive drug).

### Gut microbiota of SLE patients is altered compared to HCs

**Figure 1:**
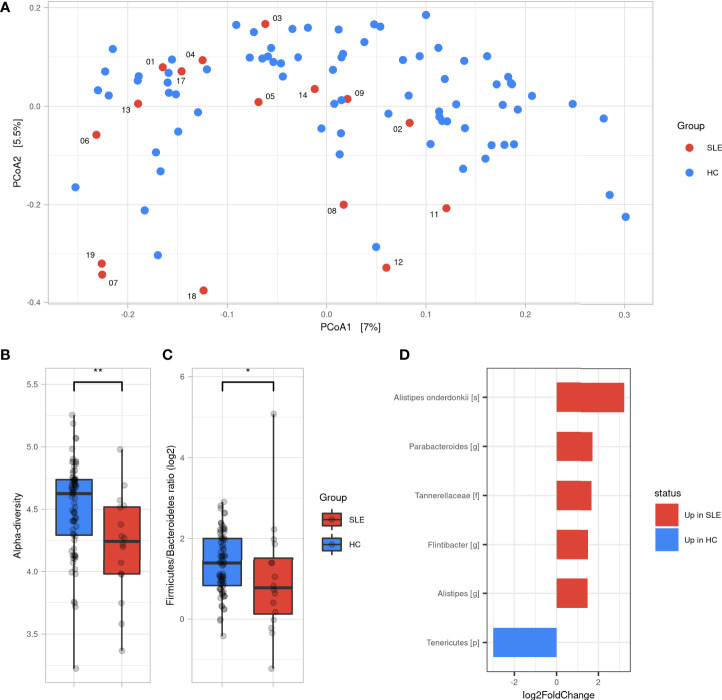
Gut microbiota difference between SLE patients and HC. **(A)** Principal coordinate analysis (PCoA) of beta-diversity based on Bray-Curtis distances. **(B)** Alpha diversity assessed by Shannon’s index between SLE and HC groups. **(C)** *Firmicutes/Bacteroidetes* ratio difference between SLE and HC groups. Statistical differences between groups are shown: *p <0.05, **p <0.01 by Wilcoxon’s test. **(D)** Differentially abundant taxa between SLE and HC groups identified by DESeq2: only taxa with adjusted p <0.05, absolute log2FoldChange >1 and prevalence per group >0.333 are shown. SLE, systemic lupus erythematosus; HC, healthy controls.

To measure the similarity of gut microbial communities’ composition, the beta-diversity was measured using Bray-Curtis distance on ASVs. A PCoA was used for visualizing samples projections and did not show clear distinct clustering pattern between SLE and HCs groups (**Figure 1A**). However, a permanova test revealed that the gut microbiota composition of SLE patients was significantly different from HCs (*p<0.01*). SLE patients showed a significant decrease in alpha-diversity compared to HCs regarding all qualitative, quantitative, and phylogenetic-aware metrics (**Figure 1B** and **Figure S1**). Moreover, a lower *F/B* ratio was observed in SLE patients (**Figure 1C**, *p<0.05*). We subsequently tracked differentially abundant taxa between SLE patients and HCs using DESeq2 to identify *de novo* biomarkers. At the phyla level, our analysis showed a significant decrease in *Tenericutes* in SLE patients (*p<0.05*). In contrast, *Tannerellaceae* family *(*p<*0.01), Alistipes (p<0.05), Flintibacter (p<0.05)* and *Parabacteroides (p<0.01)* genus were significantly abundant in SLE patients. Among *Alistipes* genus, the trend was mostly driven by one ASV that was classified as *A. onderdonkii* (*p<0.01*) and therefore this species was as well significantly more abundant in SLE patients (*p<0.001*) (**Figure 1D** and **Figure S2**).

Taken together, these findings illustrate that SLE patients have a different gut microbiota profile than HCs showing a decreased alpha-diversity and *F/B* ratio with six differentially abundant SLE biomarkers.

### Unsupervised classification reveals two distinct clusters that correlates with SLE activity

**Figure 2:**
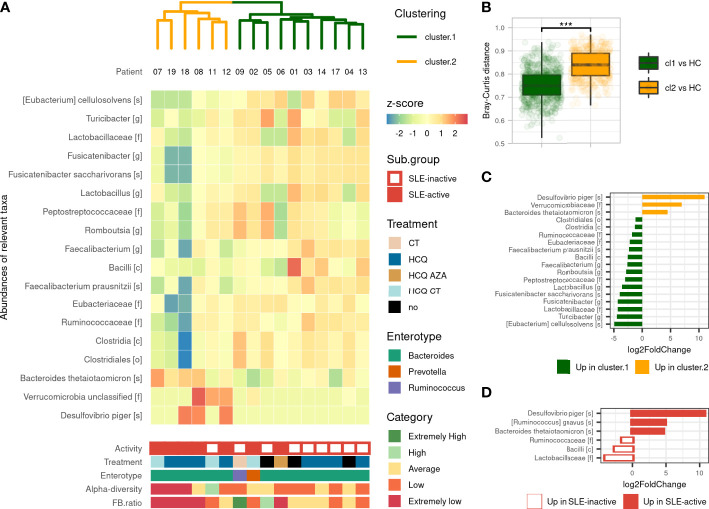
Gut microbiota’s composition-based unsupervised classification of SLE patients. **(A)** Hierarchical clustering based on Bray–Curtis and Ward’s linkage show two clusters of SLE patients based on their microbiota’s composition and Heatmap of differentially abundant taxa illustrate differences in microbiota’s composition. Activity (based on SLEDAI score), treatment and enterotype were assessed between clusters. Alpha-diversity (by Shannon’s index) and *F/B* ratio were assessed and compared to HCs distribution to evaluate for comprehensive visualization. **(B)** Pairwise Bray–Curtis distances between each patient with SLE and each HC according to clustering. Statistical difference is shown. ***p <0.001 by Wilcoxon test. **(C)** Differentially abundant taxa identified by DESeq2 between the two clusters. **(D)** Differentially abundant taxa identified by DESeq2 between active and inactive SLE patients. Only taxa with adjusted p-value <0.05, absolute log2FoldChange >1 and prevalence per group >0.333 are shown. SLE, systemic lupus erythematosus; Cl1, cluster 1; Cl2, cluster 2; CT, corticosteroids; HCQ, hydroxychloroquine; AZA, azathioprine (immunosuppressive drug).

We performed an unsupervised clustering based on gut microbiota compositions of SLE patients to look for subgroups of patients sharing similar gut microbiota. This analysis revealed two main clusters: Cluster 1 (referred to CL1) and Cluster 2 (referred to CL2), containing 10 and 6 SLE patients respectively. We observed that CL2 was enriched with active-SLE patients (Fisher’ exact test, *p<0.05*, odd ratio=15), and was composed of patients with significantly higher SLEDAI score taken as numeric value (Wilcoxon’s test, *p<0.05*). This association was not found with age, sex, BMI, treatment or enterotype excluding the possible confounding factors in the differences observed between the two clusters (**Figure 2A**). We measured the pairwise Bray-Curtis distances between each SLE patient, and each HC. We showed that CL2 patients were more distant to HCs than CL1 patients suggesting a dysbiosis gradient between the two clusters (**Figure 2B**). These alterations were subsequently observed in the *F/B* ratio, which was more disturbed in CL2 than CL1 (*p<0.05*), as well as in many differentially abundant taxa. The gut microbiota of CL2 patients was enriched with an unclassified family belonging to *Verrucomicrobia* phylum, *Desulfovibrio piger* and *Bacteroides thetaiotaomicron* species compared to CL1 patients. While some populations within the *Firmicutes* phylum were decreased including *Bacilli* class, *Clostridales* order, *Ruminococcaceae, Eubacteriaceae, Lactobacillaceae* families, *Romboutsia, Lactobacillus, Fusicatenibacter, Turicibacter* genus, *Faecalibacterium prausnitzii*, *Fusicatenibacter saccharivorans* and *Eubacterium cellulosolvens* species (**Figures 2C**). All these findings showed that our unsupervised analysis reveals two different clusters of patients with a marked dysbiosis gradient and correlated highly with their SLEDAI score suggesting that the gut microbiota is involved in the severity of the disease.

### Gut microbiota of active SLE patients is altered compared to inactive SLE patients

Given that the gut microbiota was significantly different in SLE patients compared to HCs and that the dysbiosis was highly correlated with disease activity, we next performed a supervised gut microbiota analysis between active and inactive SLE patients. We first showed that active SLE patients were more distant to HC than inactive SLE patients and consistently observed across metrics (**Figure S3**). The permanova test on beta-diversity demonstrates that the composition of active SLE patients’ microbiota was different than the one of inactive SLE patients (*p<0.001*) while no statistical difference was observed in alpha-diversity between groups. Active SLE patients have a significantly lower *F/B* ratio than inactive SLE group (*p<0.01*). Furthermore, as shown in **Figure 2D**, six differentially abundant taxa were identified including increased *Desulfovibrio piger*, *Bacteroides thetaiotaomicron* and *Ruminococcus gnavus* species and decreased *Bacilli* class, *Ruminococcaceae* and *Lactobacillaceae* families in active SLE patients compared to inactive-SLE patients. This pattern, except for *R. gnavus* species, was commonly observed in CL2 which confirms that this subgroup mainly reflects the dysbiosis that occurs in active SLE patients. Altogether, our results indicate that the gut microbiota profiling of active SLE patients were markedly different with a severe gut microbiota dysbiosis compared to inactive SLE patients.

### SLE severity signature is stable over time

To ensure the robustness of the clustering, we added to the analysis the second stool sample, available from nine of our 16 SLE patients. We noted that all samples at their second time-point where highly similar to their first time point and clustered together except for one patient as shown in **Figure S4A**. Indeed, SLE patient ‘12’ showed a dramatic change in gut microbiota composition and moved from CL2 to CL1 while no clinical changes were observed (**Table S2**). Taken together, our data shows that the severity of gut dysbiosis is stable over-time.

### PIL mouse model has a shared gut microbiota change with SLE patients

**Figure 3:**
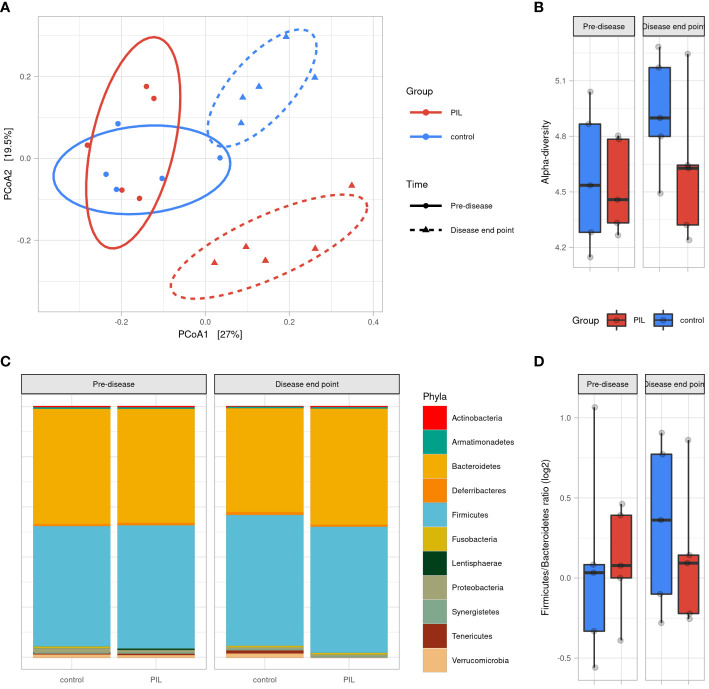
Gut microbiota difference variation overtime between PIL and Control groups. **(A)** Principal coordinate analysis (PCoA) of beta-diversitybased on Bray–Curtis distances shows that mice were uniform before induction of the disease (p = 0.6, permanova test) and strongly clustered according to groups at disease end point (6 months after induction) (p <0.01, permanova test). **(B)** Alpha diversity assessed by Shannon’s index. **(C)** Gut microbiota’s phyla composition according to groups and time point. **(D)** *Firmicutes/Bacteroidetes* ratio across groups and time points. PIL, pristane-induced lupus.

To determine the dynamics of murine gut microbiota during lupus progression, we established a PIL mouse model presenting human SLE symptoms (See **Supplementary results** and **Figure S5**). We analyzed the microbial profiles on Day 0 (pre-diseased time-point) and at M6 post-induction (diseased-endpoint). A PCoA based on Bray Curtis distance showed that the gut microbiota of CO and PIL mice were grouped together as a single pre-diseased cluster before lupus induction. Then, at the diseased endpoint, the gut microbiota split into two clusters: a cluster regrouping the CO mice and a cluster regrouping the PIL mice (*p<0.01*), suggesting a radical change in the gut microbiota during the onset of SLE-like symptoms (**Figure 3A**). No significant differences in alpha-diversity were observed between groups at baseline or at the diseased endpoint. Nevertheless, the CO mouse group had higher biodiversity at the disease end point than at baseline, while this phenomenon was not observed in the PIL mouse group (**Figure 3B**). Similarly, we did not detect a significant difference in the *F/B* ratio between the groups at any time point (**Figures 3C, D**). The taxonomical analysis revealed some bacterial population alterations in the PIL mice group at the disease endpoint compared to their pre-disease-time-point and the CO mice group. We compared the murine data only with the results of our comparison analysis between patients with SLE and HCs. Our data showed that *Tenericutes* were significantly decreased in both SLE and PIL mice. Also, the *Tannerellacea* family, *Parabacteroides*, *Bacteroides*, and *Alistipes* genera were commonly increased in PIL mice and patients with SLE (**Figure S6**). Taken together, the gut microbiota is disrupted during lupus development in PIL mice and shares five differentially abundant biomarkers with patients with SLE.

### SLE biomarkers panel proposal through existing human and murine data

**Figure 4:**
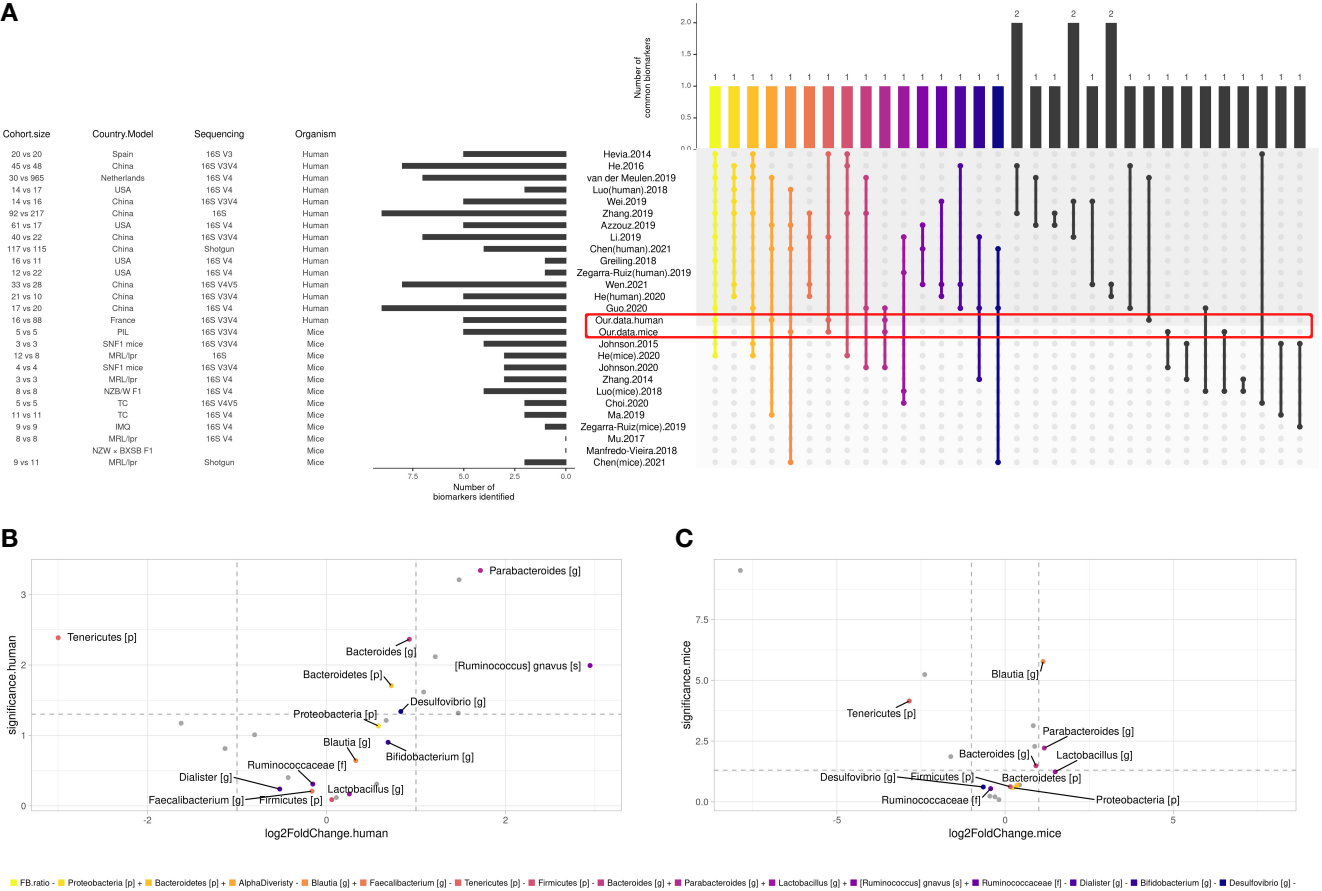
Comparison between SLE gut microbial signatures in human and mice studies across literature. **(A)** Differentially abundant taxa identified in human case-control studies and mice model. Only biomarkers identified in at least two studies are shown, and only biomarkers identified in at least three studies were attributed a color code for further investigation. **(B)** Volcano plot showing biomarkers in our human cohort and **(C)** our mice experiment, x axis shows biomarkers’ log2FoldChange and y axis shows their significance (uncorrected p-value, see ‘*Materials and methods*’).

To define a universal microbial biomarker of SLE, we performed an exhaustive literature review comparing the existing studies that have proposed an SLE gut signature compared to HCs. Among humans (n = 14) and murine studies (n = 11), 132 SLE biomarkers were identified. Biomarkers that were found in the last two studies are shown in **Figure 4A**. Overall, 16 biomarkers were commonly found in at least three studies, including decreased *F/B ratio* and alpha diversity as well as an increase in *Bacteroidetes*, *Proteobacteria* phyla, *Blautia*, *Bacteroides*, *Parabacteroides*, *Lactobacillus* genus, and *Ruminococcus gnavus* species and a decrease in *Firmicutes*, *Tenericutes* phyla, *Ruminococcaceae* family, *Faecalibacterium*, *Dialister*, *Bifidobacterium*, and *Desulfovibrio* genus. To track trends in our data sets that were not significant due to our relatively small sample size, we looked for the 16 most relevant biomarkers from the overall literature (raw p-values, no log2FoldChange cutoff). In our human data set, besides our signature, we found that *Bacteroidetes* and *Proteobacteria* phyla, *Bacteroides*, *Desulfovibrio* genus, and *Ruminococcus gnavus* species showed the same trend as the literature (**Figure 4B**). Similarly, *Bacteroides* and *Lactobacillus* genera were found coincidently in our mouse dataset (**Figure 4C**). Overall, we shared nine human biomarkers and seven murine biomarkers among the 16 biomarkers we commonly found.

**Figure 5:**
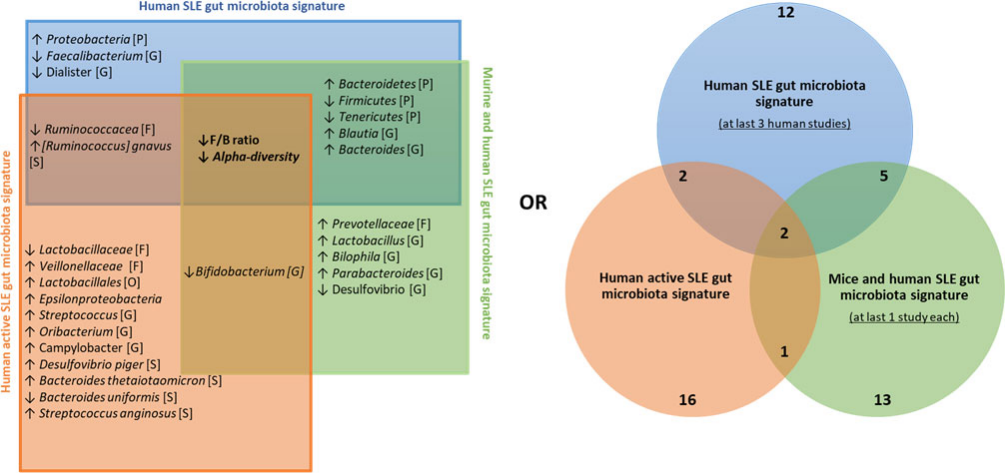
Universal panel of gut bacterial biomarkers involved in human and murine lupus and its activity. ↑, increased; ↓, decreased; [P], Phylum’s taxonomy rank; [O], order’s taxonomy rank [F], family’s taxonomy rank; [G], Genus’s taxonomy rank; [S], Species’ taxonomy rank.

We then established a universal panel of biomarkers including our finding according to i) the signature obtained in at least three human studies, ii) the common signature between human and mouse studies, and iii) the disease activity, as established by at least one study in our present work and two other studies (14, 25). **Figure 5** shows that the *F/B* ratio and alpha diversity are the core biomarkers found in every comparison. Their decrease was reported in all studies and was associated with the disease activity. Five biomarkers were commonly found in both human and murine studies including, *Bacteroidetes*, *Firmicutes*, *Tenericutes* phyla, *Blautia*, and *Bacteroides* genus. Importantly, with the contribution of our data, we report a panel of 16 biomarkers related to human disease activity. As shown in **Figure 5**, *Ruminococcaceae* and *R. gnavus* are the most identified in human studies and are related to disease activity.

## Discussion

In this study, we investigated the dynamics of gut microbiota in both human and murine lupus. We show for the first time that French patients with SLE have an imbalanced gut microbiota compared with HCs. Then, to the best of our knowledge, this study is also the first to have performed an unsupervised approach of gut microbiota in patients with SLE, irrespective of their clinical data, to investigate the correlation between the degree of their dysbiosis and their disease activity. We show that the dysbiosis of SLE gut microbiota correlates with the SLEDAI score. Thus, we propose different gut microbial signatures of human SLE according to gut dysbiosis and disease activity. In the PIL mouse model, we show a different gut microbiota composition before and after the disease onset. We further demonstrated that some bacterial populations are commonly found in patients with SLE. Based on an exhaustive cross-species comparison between our data and previous human and murine SLE studies, we propose a core-set of gut microbiome species that might constitute biomarker panels relevant for future validation studies.

In patients with SLE, an overall decrease in alpha-diversity and a reduced *F/B* ratio were observed in SLE patients. This imbalance seems to be the main feature of SLE dysbiosis, as it has been reported by almost all previous SLE cohorts independently of ethnicity, lifestyle, or disease stage (25–28). However, a lower diversity and F*/B* ratio have been associated with several other diseases such as type 2 diabetes (29), Crohn’s disease (30) or Parkinson’s disease (31), indicating that these alterations are not specific to SLE but may, however, indicate a general imbalance linked to the inflammatory process of the disease. Furthermore, a range of taxa were differentially abundant in patients with SLE compared to the HC group, including a decrease in *Tenericutes*, an increase in *Alistipes Flintibacter*, *Parabacteroides* (among *Tannerellaceae* family) genus, and *Alistipes onderdonkii* species. These gut bacteria have been implicated in health and disease in several clinical and preclinical studies. Thus, the depletion of *Tenericutes* has been previously observed in two distinct studies with active and inactive SLE patients (25, 26). These bacteria have an anti-inflammatory effect and can modulate the immune system by providing gut tolerance and preventing inflammation (32). Therefore, the increasing level of *Parabacteroides* has been previously positively correlated with inflammatory cytokines involved in SLE pathogenesis such as IL-17, IL-21, IL-2R, TWEAK, IL-35, IL-10, and IFN-γ (12) suggesting that these bacteria may play a pro-inflammatory role in stimulating immune factors. Also, *Alistipes*, a relatively recent genus of the *Bacteroidetes* phylum, was found to be increased in SLE and primary Sjögren’s syndrome American patients (28). *Alistipes* dysbiosis have been reported as harmful in anxiety, myalgic encephalomyelitis, chronic fatigue syndrome, depression, and colorectal cancer and beneficial in other diseases such as colitis, autism spectrum disorders and various fibrotic liver and cardiovascular disorders (33). These conflicting findings can be explained as the *Alistipes* genus consists of 13 different species that may have opposite effects. In our case, *Alistipes onderdonkii* was overabundant in the feces of patients with SLE. This strain was recently reported as a cause of abdominal infection (34) and is reported for the first time in SLE through our study. Our results are consistent with previous studies despite the difference in the cohort size and the geographical locations of patients. We provide further evidence that gut microbiota dysbiosis in SLE patients is characterized by an imbalance between beneficial and harmful bacteria.

Although the role of altered gut microbiota in SLE has been well established, no specific microbial signature in defining the degree of disease-related dysbiosis has yet been identified. Our unsupervised analysis of the gut microbiota in patients with SLE shows two main clusters. Clustering was strongly correlated with the SLEDAI score independently of age, sex, BMI, or enterotype of patients, excluding any other confounding factors. The gut bacterial composition of the CL1 sub-group was more similar to HCs compared to the CL2 sub-group, which was more distant, suggesting a gradient of dysbiosis between the two groups. The CL1 sub-group, with a minor dysbiosis, was mainly composed of inactive patients with SLE except for two patients. The first case (patient 02) was in the flare phase of the disease at inclusion and had become inactive one year later. The second case (patient 06) had a low SLEDAI score attributed only to his alopecia at inclusion, which may be related to stress or factors other than the disease. The CL2 sub-group, with a more severe dysbiosis, was composed of active patients with SLE except for one inactive patient with no data available to evaluate the disease progression. Therefore, we hypothesized that this patient may be progressing toward a flare phase, which could be preceded by a previous gut microbiota dysbiosis. Importantly, the severity of gut dysbiosis in the CL2 sub-group was mainly due to the disruption of certain bacterial populations that were not revealed in our comparison between patients with SLE and HCs. These include an increase in the *Verrucomicrobia* unclassified family, *Desulfovibrio piger*, and *Bacteroides thetaiotaomicron* species and a decrease in the *Bacilli* class, *Clostridales* order (under *Clostridia* class), *Ruminococcaceae*, *Eubacteriaceae*, *Lactobacillaceae* families, *Romboutsia*, *Lactobacillus*, *Fusicatenibacter*, *Turicibacter*, *Faecalibacterium* genus, *Faecalibacterium prausnitzii*, *Fusicatenibacter saccharivorans*, and *Eubacterium cellulosolvens* species.

*D. piger* has been reported as a potential gut pathobiont and have been associated with several diseases. It has been involved in the pathogenesis of inflammatory bowel disease (IBD) (35), Parkinson’s disease (36) and systemic scleroderma (37). *B. thetaiotaomicron* has been previously found in patients with SLE (28) and expressed human-anti Ro60 antibodies in the blood of patients with SLE (38), which is implicated *via* molecular mimic of Ebstein–Barr virus nuclear antigen-1 in the intuition of SLE humoral auto-immunity. In parallel, the bacterial populations that were decreased in the CL2 sub-group are all part of the *Firmicutes* phylum, which may explain the lower *F/B* ratio observed. *Firmicutes* are the main producers of butyrate, which plays a central role in the generation and maintenance of Treg cells in various gut tissues. Their decrease has been shown to be responsible for inflammatory reactions in patients with SLE. Interestingly, these bacterial populations have various beneficial roles. Among them, *F. prausnitzii* is considered one of the most important bacterial indicators of a healthy gut with anti-inflammatory effects. Its decrease has been detected in IBD, celiac disease, obesity, and diabetes (39). Similarly, the anti-inflammatory effect of *Fusicatenibacter*, particularly its *F. saccharivorans* species, has recently been demonstrated in patients and mouse models with ulcerative colitis (40) and Crohn’s disease through IL-10 induction (41, 42). As well, decreasing *Romboutsia *has recently been reported as a novel microbial biomarker for early tumor generation in cancerous mucosa (43) and Crohn’s disease (41). Also, the decrease of *Lactobacillus*, a probiotic strain which can modulate innate and adaptive immune responses, has also been previously reported in SLE but with conflicting results between studies (25, 44). In fact, *Lactobacillus* levels have been frequently correlated, positively or negatively, with other human chronic diseases (45). This genus includes many species that may play many different roles in disease pathogenesis that need to be further investigated in the future, particularly for SLE. Bacteria among the *Ruminococcaceae* family are producers of short-chain fatty acids (SCFAs), which are the main source of energy for colon cells (46) and protect the integrity of the intestinal epithelial cell membrane (47). Their decrease may lead to leaky gut. Recently, a meta-analysis of relevant research publications from around the world has shown a decreased abundance of *Ruminococcaceae* with SLE, especially in Chinese patients (48). Overall, our results show that a specific microbial signature in patients with SLE with more severe dysbiosis was found and correlated strongly with the SLEDAI activity score, suggesting the contribution of gut microbiota to the severity of the disease. These findings are supported by our supervised analysis of active and inactive patients with SLE. CL2 shared the microbial signature of active patients with SLE, including decreasing in *Bacilli*, *Ruminococcaceae*, *Lactobacillaceae*, and increasing in *D. piger*, *B. thetaiotaomicron*, suggesting that these populations are strongly associated with the severity of the disease. While *R. gnavus* was only significantly increased in active patients with SLE. Azzouz et al. have shown that the intestinal expansion of this bacteria reflects the extent of the disease activity in lupus nephritis patients (14).

Then, we investigated a potential common microbial dysbiosis signature between human and murine SLE. The PIL mouse model is characterized by typical ANA, clinical manifestations, and organ involvement similar to human SLE characteristics (49, 50), making it a relevant model for studying gut microbiota. We are the first report a gut microbiota signature in the PIL mouse model. Metagenomic data from the PIL mouse model were analyzed and found to support our findings in patients with SLE despite the small number of mice. Interestingly, five biomarkers among six were shared between patients with SLE and the PIL mouse model, including *Tenericutes*, *Tannerellaceae*, *Parabacteroides*, *Bacteroides*, and *Alistipes.* To date, only two studies have investigated both human and murine gut microbiota in SLE. Luo et al. have identified that only *Lachnospiraceae* was commonly found extended in both MRL/lpr mice and American patients with SLE (51). Then, greater consensus was commonly found between MRL/lpr and Chinese patients with SLE, including 17 species (52). In the same study, more signatures in pathway analysis were shared, including pathways of L-arginine, L-ornithine, tryptophan, and menaquinol biosynthesis that were related to SLE. More consensus is still needed between humans and mice with a larger number of patients with SLE and a mouse model to be able to continue using mice to model human disease in interventional investigations.

Our study is not without limitations. In our human cohort, the active patients with SLE have mostly mild and moderate activity. We minimized this bias through our unsupervised analysis, which was able to define patients with SLE according to their dysbiosis. It seems important to note that the exact composition of clusters may differ if samples are added or removed from the dataset and is sensitive to methodological choices, notably to distance metric and clustering linkage strategy. Also, patients with SLE were enrolled while already being diagnosed and treated. Thus, we did not investigate the impact of treatment on SLE-associated dysbiosis because we did not have their stool samples before the beginning of treatments, and the small size of our cohort precludes any definitive conclusions and warrants further studies. In our murine study, because of the unavailability of disease activity score in mice due to the small number of animals, we only compared these data to those of patients with SLE in comparison to HCs. It should also be noted that we cannot rule out the possibility that cage effects are behind the differences observed between the two groups of mice and that we do not have additional cages for each group. Longitudinal investigations in a larger number of PIL mice distributed in different cages are needed in order to establish an association between changes in the gut microbiota and the establishment of SLE at different time points. Indeed, the mechanistic link between disease susceptibility and gut microbiota changes needs to be explored in this model. Also, it remains very uncertain whether gut microbiota dysbiosis is either a causative factor or a consequence of SLE disease or both. Therefore, the identification of specific bacteria responsible for the dysbiotic state in SLE may provide a better insight into the underlying mechanism. For that proposal, several thoughtful approaches can be considered, including colonization of germ-free mice with gut bacterial populations associated with the disease, as proposed by our panel, which might offer more insight into the role of these bacteria in the disease pathogenesis.

Despite our findings, which are consistent with some existing studies, the current literature regarding a common signature of gut microbiota dysbiosis in SLE is at present ambiguous. This may be due to the lack of comparative studies between humans and mice, as discussed above, and according to the disease activity. We propose a representative pattern of gut microbiota biomarkers, which illustrates biomarker panels that are commonly found according to existing studies. Importantly, *Ruminococcaceae*, *Bifidobacterium*, and *R. gnavus* seem to play a crucial role in the severity of SLE and should be the target of future investigations to better understand the mechanisms involved. These bacterial populations are possibly trigger an auto-immune response by molecular mimicry or by influencing the Th17/Treg balance, resulting in regulatory and/or effector responses in SLE. It is a common phenomenon that a leaky bacterial product or bacteria translocation, characteristic of increased permeability of the gut, primes or educates the immune system not only in the gut but also in the entire body. Functional validation assays are needed to demonstrate the mechanistic approaches of the bacterial populations proposed in our panel and need to be enriched by other larger comparative studies.

## Data availability statement

The datasets presented in this study can be found in online repositories. The name of the repository and accession number can be found below: EBI European Nucleotide Archive; PRJEB52971.

## Ethics statement

The studies involving human participants were reviewed and approved by ANSM and CPP Nord-Ouest IV. The patients/participants provided their written informed consent to participate in this study. The animal study was reviewed and approved by the animal experimentation ethics committee under reference number APAFIS #26184.

## Author contributions

Conceptualization: LC, AP, and ET. DNA extraction and samples sequencing: ET and MB. Animal experimentation: ET and MM. Bioinformatic and statistical analyses: BG. Data interpretation: ET and BG. Writing and original manuscript preparation: ET and BG. Review and editing: LC, AP, MB, SM, and MM. Final manuscript validation: LC, AP, ET, J-LM and PH. Supervision: AP, J-LM, LC, and PH. Funding acquisition: LC and PH. All authors discussed the results and commented on the manuscript. All authors contributed to the article and approved the submission version. All authors listed have made a substantial, direct, and intellectual contribution to the work and approved it for publication.

## Funding

The study was funded by the THELLIE crowfunding plateform (https://thellie.org/lupuslivinglab) and funds from European Hospital Marseille.

## Conflict of interest

The authors declare that the research was conducted in the absence of any commercial or financial relationships that could be construed as a potential conflict of interest.

## Publisher’s note

All claims expressed in this article are solely those of the authors and do not necessarily represent those of their affiliated organizations, or those of the publisher, the editors and the reviewers. Any product that may be evaluated in this article, or claim that may be made by its manufacturer, is not guaranteed or endorsed by the publisher.

## Author disclaimer

The funders, private donors, patient association, and pharmaceutical laboratory had no role in study design, data collection and analysis, decision to publish, or preparation of the manuscript.
