# Supplementary material for: Gut microbiota in systemic lupus erythematosus patients and lupus mouse model: a cross species comparative analysis for biomarker discovery
Source: Front Immunol. 2022 Aug 2;13:943241. doi: 10.3389/fimmu.2022.943241 (PMC9378784; doi:10.3389/fimmu.2022.943241)
Supplement: Supplementary file 1 [file DataSheet_1.pdf]

Supplementary Data

1 Supplementary statistical analysis details

Alpha and Beta diversity

Alpha-diversity was assessed by Shannon index, on rarefied data, and compared when relevant to observed richness, chao1, inverse Simson and Faith's diversity as computed with picante R package (Kembel, 2020) (version 1.8.2) . *Firmicutes/Bacteroidetes (F/B)* ratio was computed on raw read counts. Differences between groups regarding alpha-diversity and *F/B* ratio was assessed by Wilcoxon's test, using 0.05 cut-off for p-values. Beta diversity was assessed by Bray-Curtis dissimilarity index, computed on rarefied data and visualized using principal coordinate analysis (PCoA). Permanova test was performed to track the effect of clinical conditions on distances between samples.

Biomarker discovery

Differentially abundant taxa, were identified using DESeq2 method (Love, 2014) (version 1.32.0) through its wrapper in microbial R package (https://CRAN.R-project.org/package=microbial) (version 0.0.20). After observing very significant p-values for very rare taxa, we post-processed the results discarding taxa that were not detected with at least 100 reads in at least one third of samples of one group of the comparison. This threshold was set to half of the samples for mice dataset, keeping only taxa that were detected in 3 out of the 5 mice of at least one group. We applied this method to each taxonomic rank (Phylum, Class, Order, Family, Genus, Species and ASVs) independently and then gathered. For easier interpretation, we did not show differentially abundant ASVs in the main figures. To ensure the consistency of microbiome signature, we considered that the taxa were differentially abundant if Benjamini-Hochberg adjusted p-values were bellow 0.05, and absolute value of log2FoldChange were greater than 1.

SLE patients' stratification

Unsupervised classification of samples was performed based on Bray-Curtis dissimilarity indices, using hierarchical clustering with Wards linkage criteria and the two main clustered were retrieved. To illustrate patient's microbiota composition related to this clustering, we displayed relevant taxa based

on differentially abundant taxa between the two clusters, as described above, with heatmap based on z-score. To do so, we retrieved relevant taxa raw number of reads, added pseudo count corresponding to half on the minimum of the resulting matrix, applied log10 transformation, and finally centered and scaled the matrix. We measured the alpha diversity and *F/B* ratio as described above and displayed the position relatively to HCs values. Values bellow 5th and above 95th percentile of HCs distribution were considered as very low and very high respectively, values between 5th and 25th percentile were labeled as low, values between 75th and 95th percentile were labeled as high, and values between 25th and 75th percentile were labeled as average. We evaluated the relation between clustering and diseases activity category and sex using Fisher's exact test on contingency table, as they were dichotomous variables. Relation between clustering and non-dichotomous qualitative variables (treatment and enterotype) were tested using chi-squared approximate test on contingency table. Relation between clustering and continuous variables (alpha diversity, *F/B* ratio, age, and BMI) were assessed with Wilcoxon's test. To confirm our visual impression on PCoA and show that microbiota of patients belonging to cluster 2 were more abnormal than the one of patients belonging to cluster 1, we measured the pairwise Bray-Curtis distances between each SLE patients, and each HCs and we tested for differences between the two clusters with Wilcoxon's test.

2 Supplementary Results

Murine mouse model validation

Before exploring the dynamic of gut microbiota in the PIL mouse model, we performed several assays to validate the onset of SLE-like-disease. The first evidence of induced autoimmunity was revealed by the presence of ANA with immunofluorescence in the sera of PIL mice compared to CO group where they were negative (**Figure S5A**). This observation was then validated by a quantitative ELISA assay of anti-dsDNA as we showed a significant increased levels of anti-dsDNA antibodies in PIL group compared to the CO group ($p<0.001$, **figure S5B**). Next, we investigated the inflammatory profile from mice sera by quantifying different pro-inflammatory cytokines. Our results showed increased levels of IFN-α, CRP and TNF-α in PIL group compared to CO group (all $p<0.0001$, **figure S5C**). Then, we demonstrated that renal manifestations have been successfully replicated in PIL model with glomerulonephritis development revealed by immune-complex deposits of IgG and C3 in the glomeruli (**Figure S5D**). PIL mouse model have also developed arthritis (**Figure S5E**) and lung damage

manifested by nodular and interstitial lymphoid infiltration with BALT hyperplasia and hemosiderin-laden macrophages (**Figure S5F**).

3 Supplementary Figures legends

Figure S1. Alpha-diversity in SLE patients and HC by different metrics. Statistical differences were shown: *p<0.05, ** p<0.01 by Wilcoxon's test. *SLE: Systemic Lupus Erythematosus, HC: Healthy controls, observed: observed richness, InvSimpson: Inverse Simpson, PhyloDiversity: Faith's phylogenetic diversity.*

Figure S2. Prevalence and relative abundance of differentially abundant taxa identified by DESeq2. *SLE: Systemic Lupus Erythematosus, HC: Healthy controls.*

Figure S3. Pairwise distances between each SLE patient and each HC according to disease activity. Statistical differences are shown. *** p<0.001 by Wilcoxon test. *SLE: Systemic Lupus Erythematosus, HC: Healthy controls.*

Figure S4. Longitudinal gut microbiota analysis of SLE patients. (A). Principal coordinate analysis (PCoA) based on Bray-Curtis distance. Dot lines connect two-time point for the same SLE patient. (**B**) Hierarchical clustering based on Bray-Curtis and Ward's linkage that include both time-point when available.

Figure S5. Lupus mouse model validation at 6 months after pristane induction compared to control group. (A) Auto-immunity evaluation represented by photomicrographs (x20) of anti-nuclear antibodies (ANA) in control and pristane-induced lupus mice (PIL) determined by immunofluorescence staining on Hep-2 cells. **(B)** Quantification of anti-double stranded DNA (anti-DNA) antibodies by enzyme-linked immunosorbent assay (ELISA) and expressed in ng/ml. **(C)** Inflammatory process evaluation by ELISA quantification of interferon (IFN)-α cytokine expressed in pg/ml, C-reactive protein (CRP) cytokine concentration expressed in ng/ml and tumor necrosis factor (TNF)-α cytokine expressed in pg/ml. Significant differences were observed between the groups and

p value are shown: * *p<0.05, **p <0.01* using *unpaired t-test.* **(D)** Immune-complex microphotographs of direct immunofluorescence showing a mesangial deposit of IgG and C3 in PIL mice. **(E)** Macroscopic evaluation of paws joints showing the presence of arthritis in PIL group manifested by swelling and redness (black arrows). **(F)** Representative histopathological sections of lungs in HPS stain (x180) showing a nodular and interstitial lymphoid infiltration with BALT hyperplasia (blue arrow) and haemosiderin-laden macrophages (brown arrow) in PIL mice.

Figure S6. Gut microbiota's genera composition according to groups and time point in PIL and control mice groups. Only top 15 genus are shown**.** *PIL: pristane-induced lupus*.

Figure S7. Differentially abundant taxa between PIL mice and controls. Volcano plot representing differentially abundant taxa as identified by DESeq2 at baseline (**A**) and disease end point (**B**), as well as log2FoldChanges of these taxa at disease and point (**C**). Only taxa with adjusted p-value < 0.05, absolute log2FoldChange > 1 and prevalence per group > 0.5 are shown.

4 Supplementary tables

Table S1. Characteristics of SLE patients at their second sampling

PGA: Physician Global Assessment; SLEDAI: SLE Disease Activity Index; AHT: Arterial hypertension; APS: antiphospholipid syndrome; HCQ: hydroxychloroquine; CT: corticosteroids; AZA: azathioprine (immunosuppressive drug).

Patient no.	PGA	SLEDAI	Low complement levels	Positive anti-dsDNA titres	AHT	Type 2 diabetes	APS	Ongoing SLE treatments
**001**	0.18	6	yes	yes	no	no	no	no
**002**	0.3	0	no	no	no	no	no	HCQ CT
**003**	0.15	0	no	no	no	no	yes	HCQ
**004**	0	0	no	no	no	no	no	no
**005**	0.6	4	no	no	no	no	no	no
**006**	0.15	0	no	no	no	no	yes	HCQ AZA
**007**	-	-	-	-	-	-	-	-
**008**	-	-	-	-	-	-	-	-
**009**	0.21	0	no	no	no	no	no	CT
**011**	-	-	-	-	-	-	-	-
**012**	0.27	4	yes	yes	no	no	no	HCQ
**013**	0	2	yes	no	no	no	no	HCQ
**014**	-	-	-	-	-	-	-	-
**017**	-	-	-	-	-	-	-	-
**018**	1.41	2	no	no	no	yes	no	HCQ
**019**	-	-	-	-	-	-	-	-

References

Love, M. I., Huber, W., and Anders, S. (2014). Moderated estimation of fold change and dispersion for RNA-seq data with DESeq2. *Genome Biology* 15, 550. doi: 10.1186/s13059-014-0550-8.

Kembel, S. W., Cowan, P. D., Helmus, M. R., Cornwell, W. K., Morlon, H., Ackerly, D. D., et al. (2010). Picante: R tools for integrating phylogenies and ecology. Bioinformatics, 26(11), 1463-1464. doi: 10.1093/bioinformatics/btq166.
