# Supplementary figures and images for: Gut microbiota in systemic lupus erythematosus patients and lupus mouse model: a cross species comparative analysis for biomarker discovery
Source: Front Immunol. 2022 Aug 2;13:943241. doi: 10.3389/fimmu.2022.943241 (PMC9378784; doi:10.3389/fimmu.2022.943241)

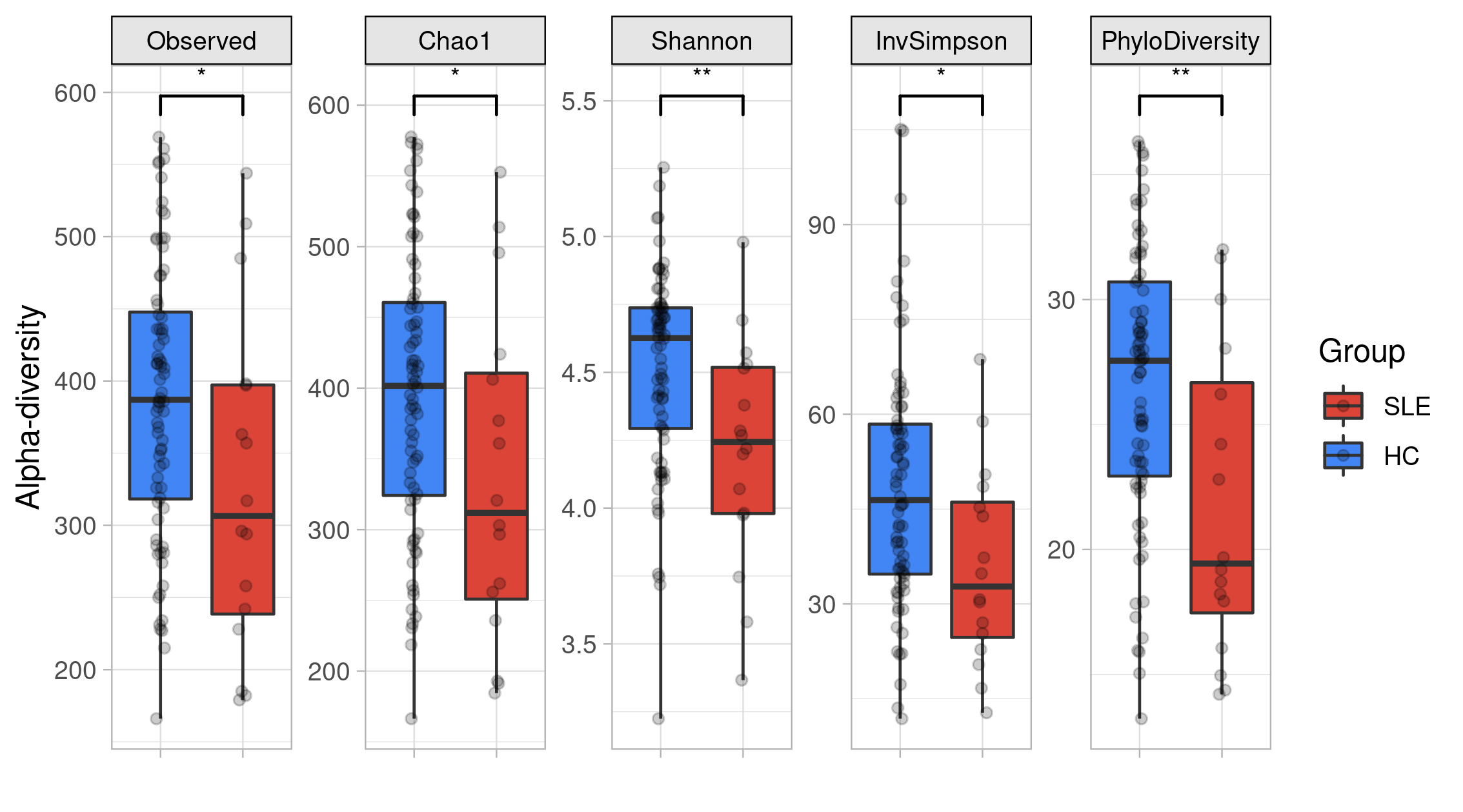

Supplement: Supplementary file 2 [file Image_1.tiff]

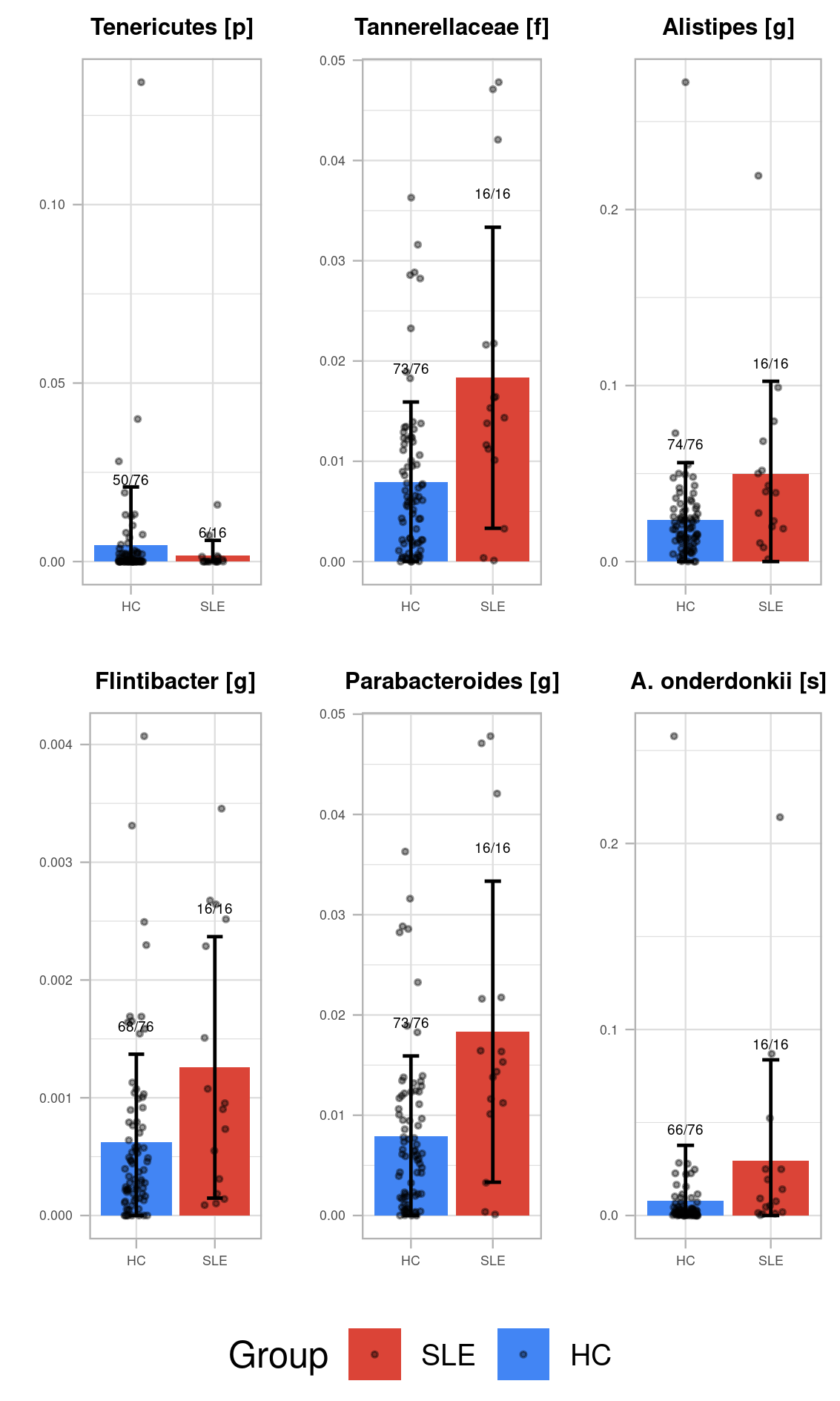

Supplement: Supplementary file 3 [file Image_2.tiff]

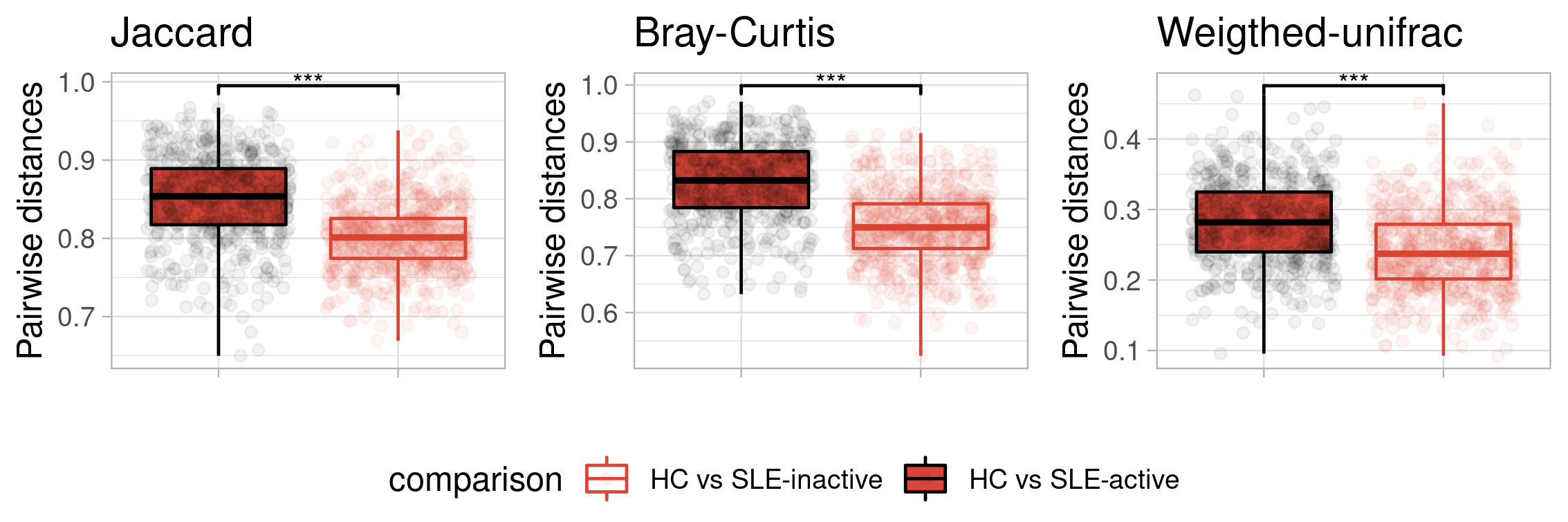

Supplement: Supplementary file 4 [file Image_3.tiff]

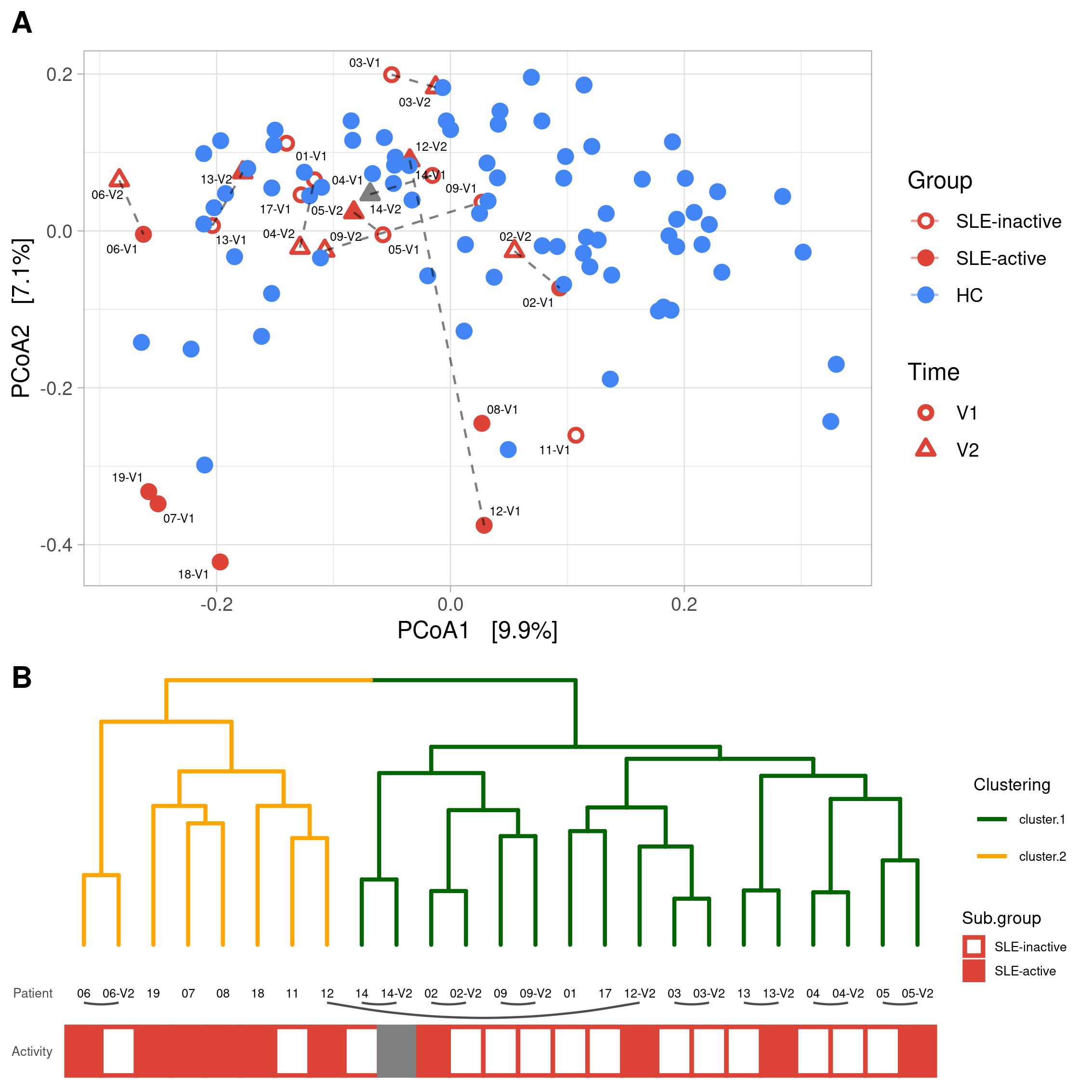

Supplement: Supplementary file 5 [file Image_4.tiff]

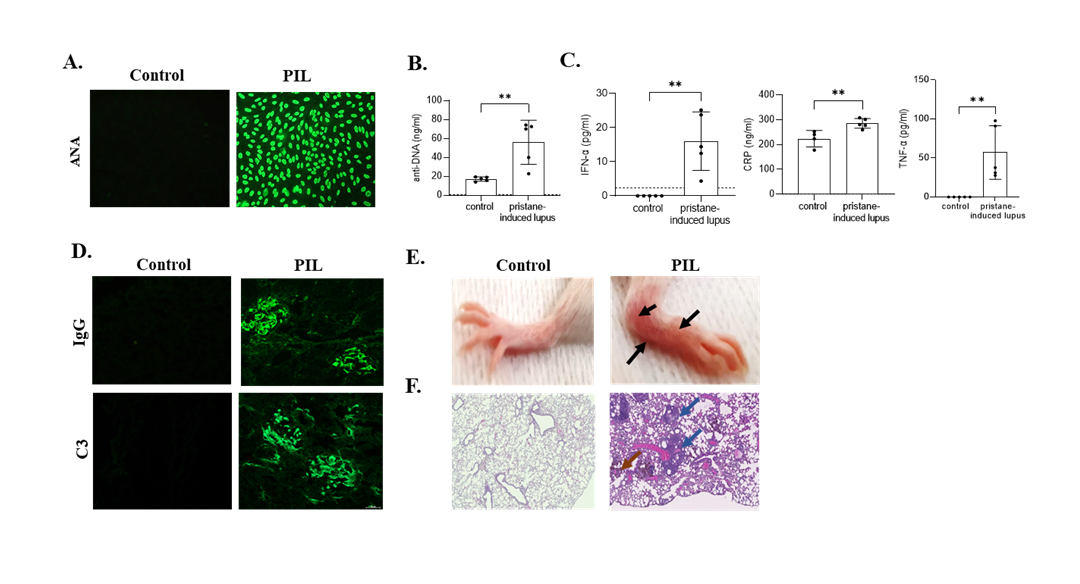

Supplement: Supplementary file 6 [file Image_5.tif]

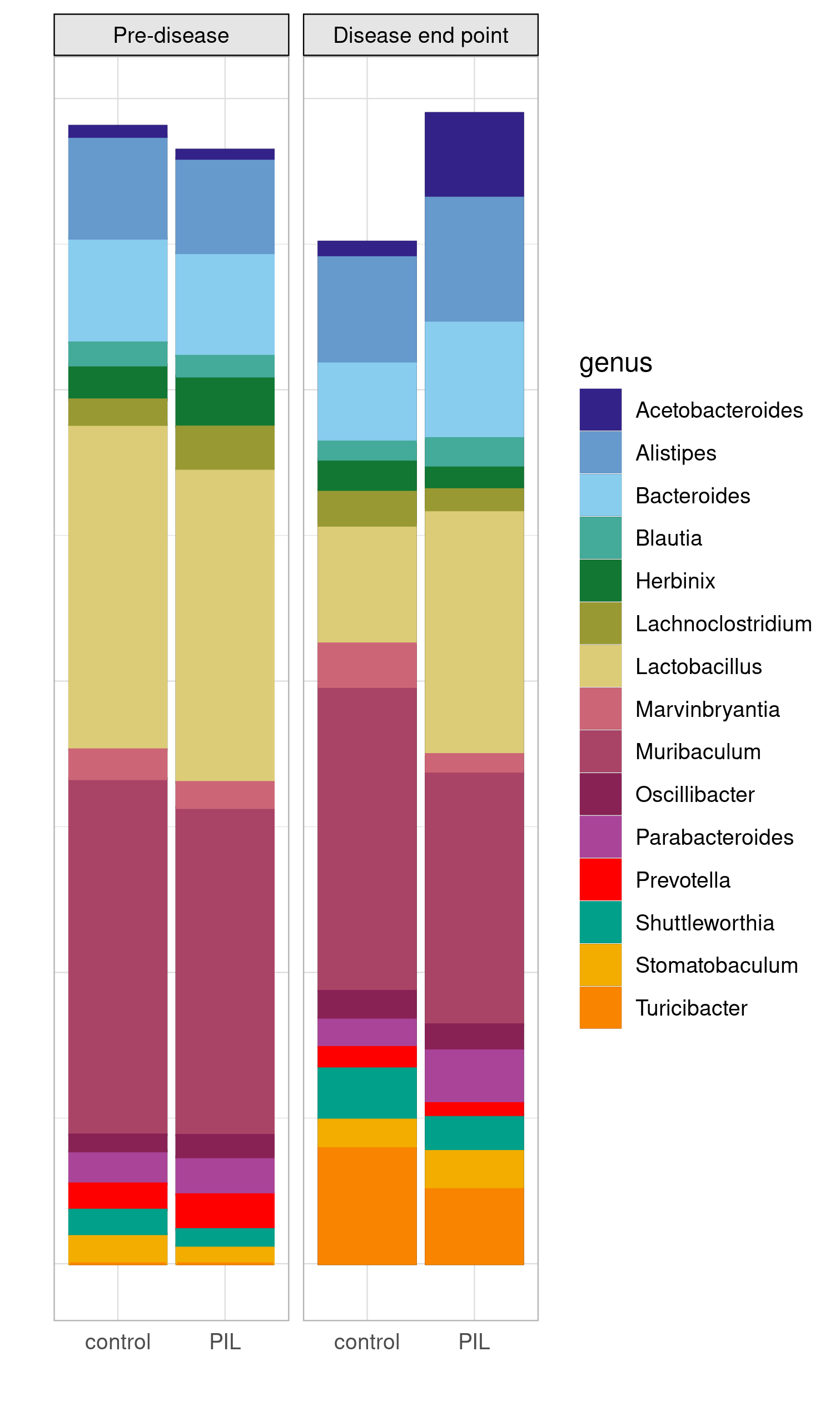

Supplement: Supplementary file 7 [file Image_6.tiff]

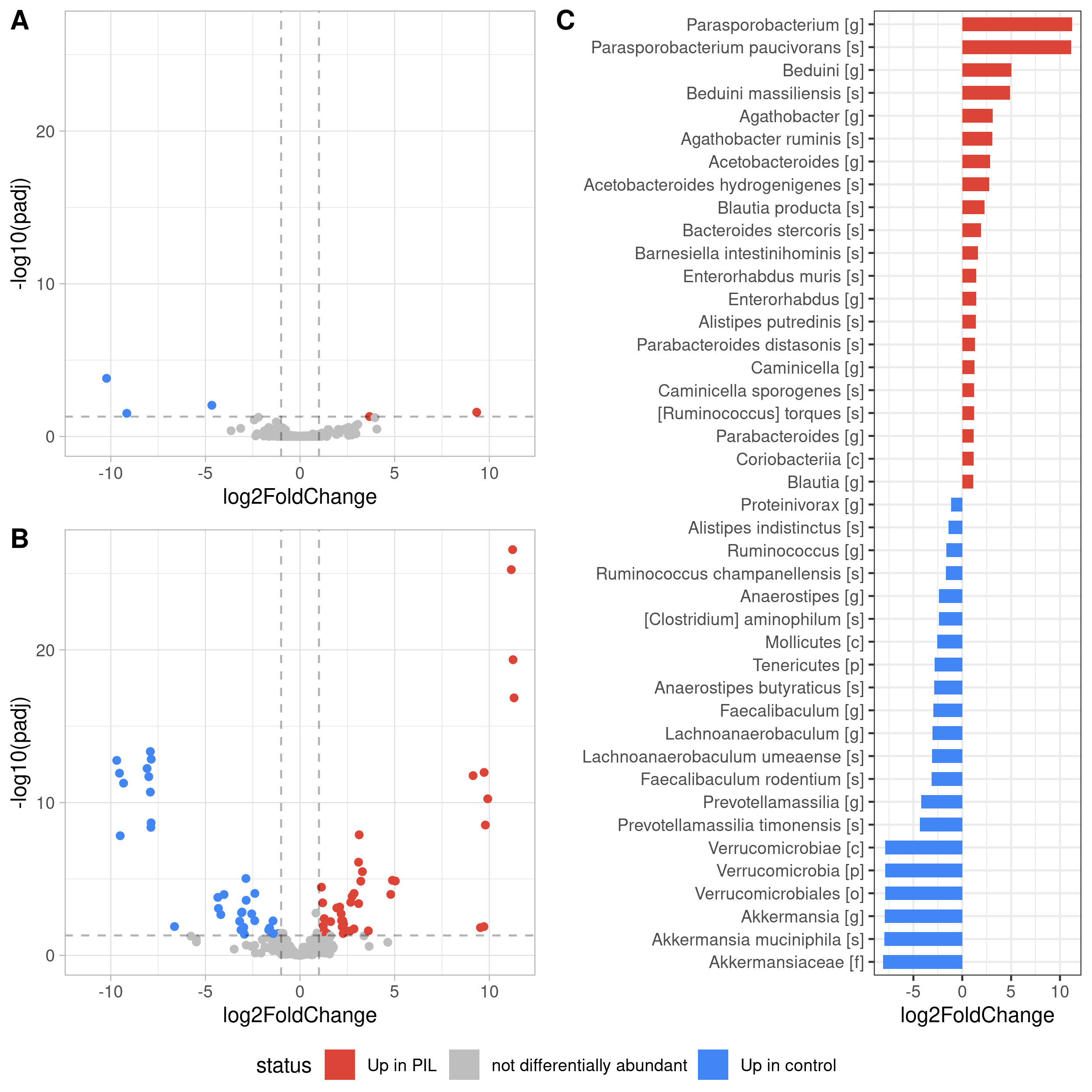

Supplement: Supplementary file 8 [file Image_7.tiff]
